# Reduced microbe abundance in an urban larval development container increases *Aedes aegypti* susceptibility to Zika virus

**DOI:** 10.1371/journal.ppat.1013154

**Published:** 2025-05-19

**Authors:** Margaret V. Becker, Anastasia Accoti, Angel Elma I. Abu, Julia Vulcan, Massamba Sylla, Kamil Khanipov, Laura B. Dickson

**Affiliations:** 1 Department of Microbiology and Immunology, University of Texas Medical Branch, Galveston, Texas, United States of America; 2 Laboratory Vectors & Parasites, Department of Livestock Sciences and Techniques, Sine Saloum University El Hadji Ibrahima NIASS, Kaffrine Campus, Karffrine, Senegal; 3 Department of Pharmacology and Toxicology, University of Texas Medical Branch, Galveston, Texas, United States of America; 4 Center for Vector-borne and Zoonotic Diseases, University of Texas Medical Branch, Galveston, Texas, United States of America; 5 The West African Center for Emerging Infectious Diseases, Centers for Research in Emerging Infectious Diseases, Galveston, Texas, United States of America; 6 Institute for Human Infections and Immunity, University of Texas Medical Branch, Galveston, Texas, United States of America; Pennsylvania State University - Main Campus: The Pennsylvania State University - University Park Campus, UNITED STATES OF AMERICA

## Abstract

*Aedes aegypti* mosquitoes are a major vector of arboviruses that oviposit in both artificial containers (i.e., buckets, tires, cans) and natural containers (i.e., coconut husks, tree holes). These diverse container types will seed the larvae microbiome with differing bacterial communities. While the larval microbiome has been shown to alter adult susceptibility to arboviruses including dengue (DENV) and Zika virus (ZIKV), it is not known if exposure to different bacterial communities found between container types impacts adult *Ae. aegypti* interactions with arboviruses. To address this, rainwater was collected from an artificial container (plastic buckets) and a natural container (coconut husks) from three different collection sites and the microbiomes were preserved. Larval exposure to plastic bucket-derived microbiomes resulted in adults with increased susceptibility to ZIKV compared to larval exposure to coconut husk-derived microbiomes from all three collection sites, indicating that the container type, independent of collection environment, drives variation in adult susceptibility to ZIKV. 16S amplicon sequencing of larvae exposed to the preserved microbiomes revealed that bacterial community structure differed between plastic bucket and coconut husk derived communities at each collection site, but a conserved plastic- or coconut-derived bacterial community across collection sites was not identified. However, water from coconut husks had significantly more total bacterial abundance than water from plastic buckets. Normalization of bacterial loads between container types resulted in similar ZIKV infection rates. Together, these data suggest that larval exposure to specific container type-associated microbiomes alters adult susceptibility to ZIKV, largely driven by differences in total bacterial density between container types. Results from this study will help understand how the urbanization-driven expansion of *Ae. aegypti* into new/different oviposition sites might affect arbovirus susceptibility.

## Author summary

*Aedes aegypti* mosquitoes, which spread viruses like dengue and Zika, inhabit different environments within Africa. Ancestral forms of *Ae. aegypti* occupy forested habitats, while domesticated forms of *Ae. aegypti* have adapted to thrive in urban environments in close proximity to humans. Increasing urbanization in Africa will likely push *Ae. aegypti* from the forested environments into more urbanized environments, necessitating a better understanding how variation between these environments can facilitate viral emergence or transmission. A source of environmental variation between forest and urban habitats is the larval development containers, which contain diverse microbial communities. In this study, we demonstrate that larval exposure to a microbial community preserved from representative urban container (plastic bucket) alters Zika virus infection in mosquitoes compared to larval exposure to microbial community preserved from representative forest container (coconut husk). Although further work needs to be done to extend these results across the entirety of urban and forest containers that *Ae. aegypti* use, these results provide empirical evidence that container type shapes the larval bacterial community and drives variation in Zika virus infection indicating that urbanization and the spread of *Aedes aegypti* into new breeding sites could affect its susceptibility to virus.

## Introduction

The *Aedes aegypti* mosquito is a vector of numerous arthropod-borne viruses (arboviruses) including dengue (DENV), yellow fever (YFV), Zika (ZIKV), and Chikungunya viruses (CHIKV), and can be found all over the world [1,2]. Climate models all predict the expansion of *Ae. aegypti* into new environments increasing the risk of arboviral diseases spread by this vector [3,4]. Alongside changing environments, global urbanization is expected to continue [5], which could also facilitate the expansion of *Ae. aegypti* distribution, specifically within Africa where *Ae. aegypti* persists across a range of urbanization. Arbovirus infections and outbreaks are driven by domesticated forms of *Ae. aegypti* specialized to live in close contact with humans in urban environments [6–8]. This is in contrast to the ancestral form that inhabits sylvatic environments and does not contribute to outbreaks. The growing human population in Africa is expected to double by 2050 [3], two-thirds of which will be absorbed by urban areas [9]. Large urban centers facilitate outbreaks and epidemics of arboviruses, which will likely shift the disease burden from malaria to arboviruses in Africa [10].

While all life stages are impacted by environmental differences between urban and sylvatic habitats, differences in larval habits between the two environments are diverse. Sylvatic oviposition sites include natural containers such as tree holes, fruit husks, and rock pools. Urban oviposition sites consist of human-associated containers like tires, cans, and plastic buckets. *Ae. aegypti* larvae acquire their microbiome from the water within their larval habitat [11–16]. Previous work has demonstrated that the microbiome within natural larval habitats in the field varies between urban and sylvatic environments [15,17]. Specifically, urban and sylvatic sites have differences in the diversity of bacterial species present and bacterial compositions [15]. Additionally, urban larval containers have lower bacterial abundance than sylvatic containers [17]. Taken together, these findings indicate that larvae developing in urban containers are exposed to different microbiomes compared to larvae developing in sylvatic containers.

Along with the adult microbiome [18–22] the larval microbiome also drives variation in *Ae. aegypti* interactions with arboviruses [15,23,24]. Exposure to different bacterial isolates or communities during larval development in a gnotobiotic system can produce adults with differences in dengue virus dissemination titers [15] and susceptibility to ZIKV [25]. Additionally, influence of specific larval microbiomes on adult ZIKV susceptibility is dependent on the mosquito genotype [25]. The diversity of bacterial species in larval water has also been shown to impact ZIKV susceptibility in adults [24]. While these studies demonstrated that the larval microbiome has a carry-over effect on the adult arboviral susceptibility, it remains unknown whether a conserved bacterial community is present across container types that correlates with a predictable differences in arbovirus infection.unknown whether a conserved bacterial community is present across container types that correlates to a predictable infection phenotype.

Given that bacterial communities differ between urban and sylvatic larval habitats and that urbanization will likely drive *Ae. aegypti* into more urban areas where they will exploit more urban oviposition containers, we sought to expand on previous work [15,25] to determine if container-specific differences in the larval microbiome result in predictable impacts on adult susceptibility to a representative arbovirus (ZIKV). First, we characterized the susceptibility to ZIKV in adults exposed at the larval stage to microbiomes preserved from rainwater collected from either a plastic bucket or a bamboo shoot. An increased susceptibility in the plastic bucket group justified expanding to include additional sites for rainwater collection. Microbiomes were preserved from replicate plastic buckets and coconut husks at three collection sites. Larvae exposed to the preserved plastic bucket microbiomes produced adults with increased disseminated ZIKV infection rates compared to larvae exposed to the coconut husk-derived (coconut-derived) microbiomes across all three collection sites suggesting that the container type microbiomes were a predictor of infection. Next, we characterized the bacterial community structure in the larvae following exposure to microbiomes from the different container types and collection sites which revealed that larval bacterial community structure was dependent on the container type within each collection site, but a conserved plastic- or coconut- derived bacterial community across collection sites was not identified. Interestingly, the coconut-derived microbiomes had a higher bacterial abundance. When the amount of bacteria in the coconut-derived community was normalized to the bacterial load in the plastic bucket-derived (plastic-derived) microbiome, similar ZIKV disseminated infection rates indicating that larval exposure to different quantities of bacteria, as determined by container type, can influence adult ZIKV susceptibility independent of bacterial species present.

## Results

### Larval exposure to a plastic bucket-derived microbiome increases ZIKV infection rates

**Fig 1:**
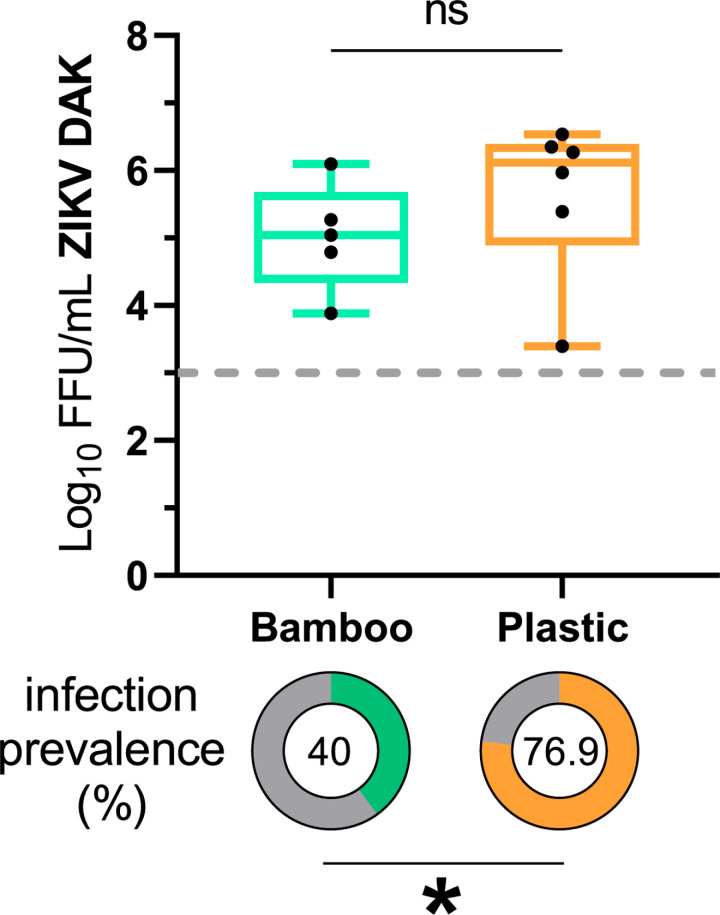
Larvae reared with a plastic bucket-derived microbiome produce adults with altered ZIKV susceptibility. The proportion of infected *Ae. aegypti* (KED colony) bodies and the amount of infectious virus in *Ae. aegypti* heads exposed to ZIKV Dakar (41524) after larval development in either a bamboo shoot-derived microbiome or a plastic bucket-derived microbiome. Bodies and heads were collected 14 days post-infectious bloodmeal. Infection prevalence and viral titers were determined using a ZIKV-specific PCR and focus-forming unit assay respectively. The boxplot shows the disseminated titers of infectious ZIKV particles expressed as the Log_10_-transformed number of focus-forming units (FFU) per ml detected in the *Ae. aegypti* heads. Each point represents an individual head, and a horizontal line represents the mean. The error bars represent the min and max. Data were analyzed by unpaired T-test as a function of titer (Bamboo vs. Plastic: p-value = 0.3319). Only positive individuals are represented (Bamboo: n = 5; Plastic: n = 6). The pie charts show the proportion of ZIKV-infected bodies which were compared by a Chi-square analysis of a 2 X 2 contingency table as a function of infection (Bamboo vs. Plastic: p-value = 0.0374). The total number of individuals tested in the Bamboo group was 20 and 13 for the Plastic group. Data represent one experiment.

Larval exposure to different complex bacterial communities influences adult *Ae. aegypti* susceptibility to ZIKV [24,25]. To determine if larval exposure to microbiomes collected from different containers in the same environment resulted in variation in adult susceptibility to ZIKV, *Ae. aegypti* larvae (KED line) were reared in the presence of microbiomes preserved from a bamboo shoot and a plastic bucket in a gnotobiotic system. Rainwater was collected from bamboo shoots and a plastic bucket and returned to the laboratory where glycerol stocks were made to preserve the microbiome (May and August 2021 collections, see materials and methods) which has been demonstrated as a viable method to preserve and store bacterial communities in larval containers [26,27]. Given the amount that this bacterial source is diluted by sterile water to provide enough volume for larval rearing in the cell culture flask, we anticipated that any effects observed would be due to the bacteria/microbiome introduced, rather than other factors such as any chemicals or minerals deposited in the container from the environment. To confirm that different microbiomes were introduced in the gnotobiotic system, 16S amplicons were compared in L3/L4 larvae. Larvae reared with the bamboo shoot-derived (bamboo-derived) microbiome had higher levels of alpha diversity (Welch t-test, p-value > 0.0001) (S1A Fig). Principal component analysis (PCoA) on a Bray-Curtis dissimilarity matrix demonstrated that the population structure differed between the two treatment groups (PERMANOVA, p-value = 0.001) (S1B Fig). Following gnotobiotic exposure to bamboo- or plastic-derived microbiomes, adults were challenged with ZIKV (Dakar strain) (S1 Table). Larval exposure to plastic-derived microbiomes resulted in a 70% infection rate in the body compared to 40% for those reared in bamboo-derived microbiomes (Chi-square on a 2 X 2 contingency table, p-value = 0.0374) (Fig 1A). These differences in infection rates did not extend to detectable differences in the number of virus particles in the head (unpaired t-test, p-value = 0.3319) (Fig 1B), a proxy for transmission potential [28–30].

### Larval exposure to plastic bucket-derived microbiomes increases ZIKV infection rates across environments

**Fig 2:**
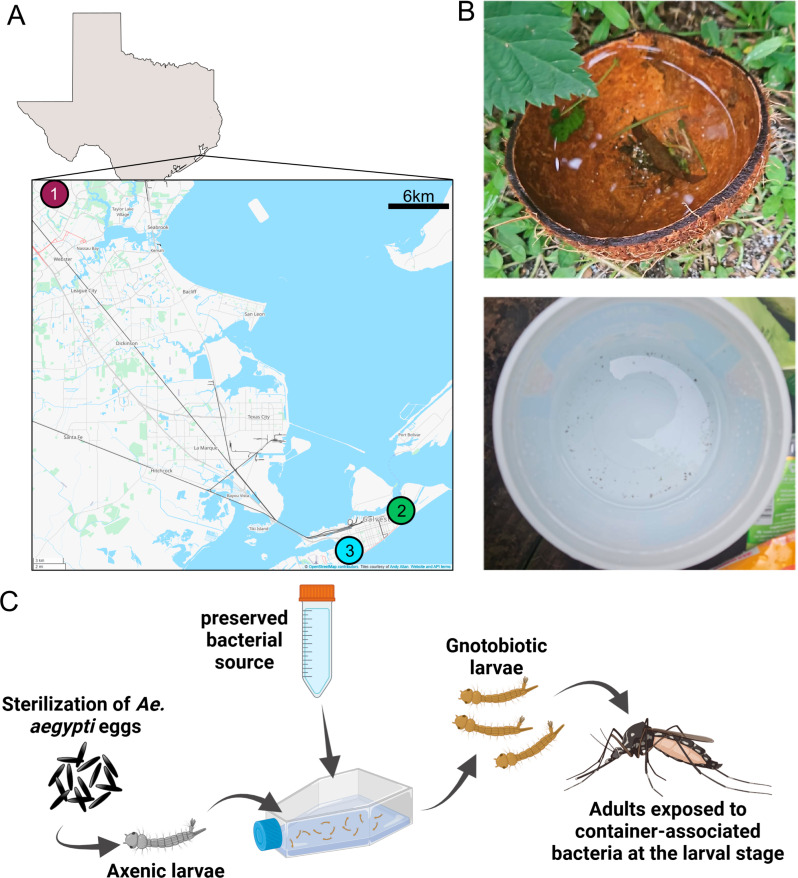
Experimental workflow. (A) Water was collected from mock oviposition containers at the three locations marked on the map. Base map and data from OpenStreetMap and OpenStreetMap Foundation. https://www.openstreetmap.org/export#map=12/29.4320/-94.9372&layers=T. Copyright: https://www.openstreetmap.org/copyright. OpenStreetMap is *open data*, licensed under the Open Data Commons Open Database License (ODbL) by the OpenStreetMap Foundation (OSMF). (B) Mock oviposition containers at each collection site included a coconut husk (top photo) and a plastic bucket (bottom photo). At each collection site, water was collected into sterile glass bottles using a sterile funnel. The water samples were brought to the lab where the bacteria were preserved with glycerol. (C) For functional assays *in vivo*, gnotobiotic larvae were created by adding the preserved bacteria to sterile flasks containing axenic larvae. Adult mosquitoes that had undergone different gnotobiotic treatments as larvae were used to test for susceptibility to ZIKV. Figure created in BioRender Becker, M. (2025) https://BioRender.com/4yx0jvm. Photos used with the permission of photographer, Dr Anastasia Accoti.

Given that we observed differences in ZIKV infection rates following larval exposure to microbiomes derived from different container types in the same environment, we sought to determine if we could detect similar differences across multiple environments (collection sites). Microbiomes were preserved from rainwater collected from replicate containers in three locations, two in Galveston, TX, and one in Houston, TX (Fig 2A) (S2 Table). Containers were placed either near the residence (urban-derived container – plastic bucket) or near foliage (sylvatic-derived container – coconut husks) (Fig 2B) with at least 5 meters between containers for one week to be seeded by different environmental bacteria (May 2023 collection, see materials and methods). Coconut husks were used instead of bamboo shoots due to the low water volume obtained from the bamboo shoots in the previous experiment. *Ae. aegypti* larvae (PKT line, similar genetic background to KED) were exposed to either the coconut husk and the plastic bucket collected water in the gnotobiotic system. To identify any fitness costs of the introduced microbiomes, pupation rates were calculated by counting the total number of pupae divided by the total number of larvae recorded each day (S2 Fig). There was no significant difference observed between the PD_50_ of the gnotobiotic larvae across the different bacterial sources (One-way ANOVA, p-value = 0.9108) (S2 Fig).

**Fig 3:**
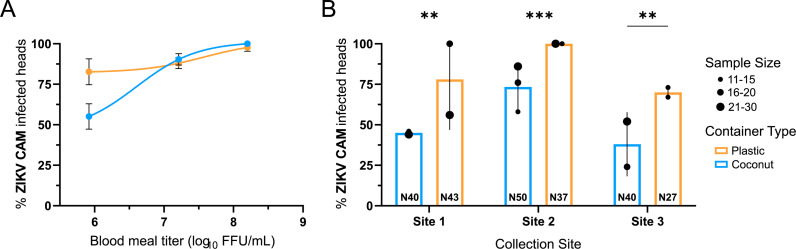
Disseminated ZIKV infection for adults from larvae reared in coconut- or plastic-derived bacteria. The proportion of infected *Ae. aegypti* (PKT line) heads exposed to ZIKV after larval development in a coconut-derived microbiome or a plastic-derived microbiome across three collection sites. Heads were collected fourteen days post-infectious bloodmeal. Proportion infected was determined using a focus forming unit assay. (A) Dose-response curve across titers of ZIKV Cambodia (FSS13025). Data represent all three collection sites merged by container from up to three independent replicates. (B) Bar graph showing the proportion of ZIKV-infected heads for each collection site at the lowest bloodmeal titer. The proportion of infected heads was compared by a Chi-square analysis of a 2 X 2 contingency table as a function of infection (Site 1: Coconut vs. Plastic: p-value = 0.0062; Site 2: Coconut vs. Plastic: p-value = 0.0013; Site 3: Coconut vs. Plastic: p-value = 0.0146). The error bars represent SEM. Data represent up to three independent replicates as indicated by the black dots. The size of the black dot represents the number of individuals per independent experiment. The total number of individuals for each group tested is displayed at the bottom of the bar.

To determine if larval exposure to the introduced microbiomes impacts adult ZIKV infection rates, adults emerging from the gnotobiotic system were exposed to three different infectious doses of ZIKV (Cambodia) (S1 Table). No differences in infection rates were detected in the head following exposure to the two highest titers, but individuals exposed to the plastic-derived microbiome resulted in a higher proportion of infected heads at the lowest inoculation titer (Fig 3A). To determine if this phenotype is consistent across the three environments, samples were split and analyzed by both collection site and container type (Fig 3B). At the low titer, differences in infection rates were driven by container type (Chi-square on GLM, p-value < 0.001) and the environment (Chi-square on GLM, p-value < 0.001), but not the interaction between container type and environment (Chi-square on GLM, p-value = 0.086). When analyzed within each collection site, the adults exposed to the plastic-derived microbiome were more susceptible than those exposed to the coconut-derived microbiome (Chi-square on 2 X 2 contingency table; Coconut vs. Plastic - Site 1: p-value = 0.0062; Site 2: p-value = 0.0013; Site 3: p-value = 0.0146). As we observed previously, no significant difference in the number of infectious virus particles replicating in the head was detected between containers or sites but we did observe a difference in the interaction between container type and site. (Two-way ANOVA, container type: p-value = 0.2503, collection site: p-value = 0.8849, container type x collection site: p-value = 0.0160)(S3 Fig).

### Both container type and environment determine the larval microbiome

**Fig 4:**
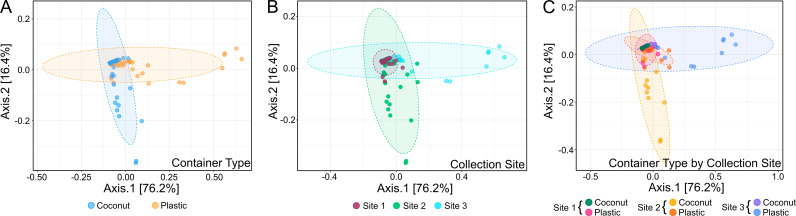
The bacterial community structure differs between gnotobiotic larvae reared in plastic and coconut water sources. The structure of bacterial communities was determined by deep sequencing the V3-V4 region of the bacterial 16S gene in individual larvae reared in either coconut-derived or plastic-derived water. Bacterial structure is represented by principal component analysis of a Bray-Curtis dissimilarity matrix based on mean genera abundance by (A) container type, (B) collection site, and (C) container type and collection site. P-values from PERMANOVA analysis are as follows (A) p-value = 0.003, (B) p-value = 0.001, (C) p-value = 0.001.

To confirm that we had introduced distinct bacterial communities into the gnotobiotic system, the bacterial composition of the input microbiome was characterized by 16S amplicon sequencing. Principal component analysis (PCoA) on a Bray-Curtis dissimilarity matrix demonstrates that the bacterial community structure differed between the preserved water sources and largely clustered by container type (PERMANOVA, p-value = 0.001) (S4 Fig), indicating that microbiomes collected from plastic buckets represent different bacterial communities than those collected from coconut husks regardless of collection site. To determine whether this is maintained in the larvae, we characterized the bacterial communities of the gnotobiotic larvae reared with preserved water from each container type/collection site. Out of 91 individual larvae sequenced, a total of 65 OTUs were found, which represent 14 genera after filtering for low abundance OTUs and OTUs present in the negative controls. Rarefaction curves showed that sufficient sequencing depth was achieved (S5 Fig). The OTU richness measured with Chao1 was similar across collection sites and container type (ANOVA, p-value = 0.26344) (S6 Fig). Larvae exposed to the coconut-derived microbiomes had distinct bacterial communities from larvae exposed to plastic-derived microbiomes (**PERMANOVA**, p-value = 0.001) (Fig 4A) and this was true across the three collection sites (S7A–7C Fig). Additionally, larvae exposed to microbiomes from each site had different bacteria community structures (**PERMANOVA, p-value = 0.001**) (Fig 4B) and this was true both within larvae exposed to the coconut-derived and plastic-derived microbiomes (S7E and S7F Fig). Despite differences between container types and between collection sites when analyzed independently, the overall bacterial community structures did not cluster by container type or collection site when data was analyzed together. Instead, the bacterial community structures were similar amongst the collection sites and container types, with the exception of the larvae exposed to the plastic-derived microbiome from Site 3 and the larvae exposed to the coconut-derived microbiome from Site 2 (PERMANOVA, p-value = 0.001) (Fig 4C).

**Fig 5:**
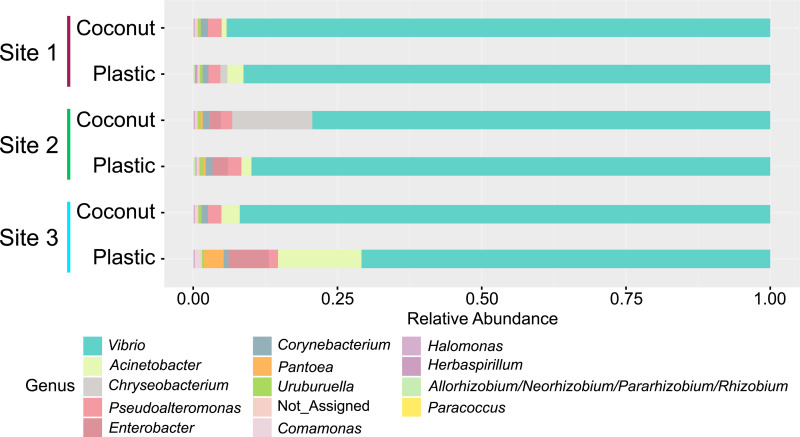
Abundance of specific taxa bears similarities between gnotobiotic larvae reared in plastic and coconut water sources. The percent abundance of the genera is plotted by collection site (Site 1-3) and container type (coconut or plastic).

To assess whether larvae acquired different bacterial taxa after exposure to different container-derived bacterial communities, the percent abundance of different genera was compared among container types and collection sites. The relative abundance of genera was similar between the container types and collection sites except for the larvae exposed to the plastic-derived microbiome from Site 3 and the larvae exposed to the coconut-derived microbiome from Site 2 (Fig 5). Additionally, the abundance of specific genera was consistent among individuals from each treatment group (S8 Fig).

To identify differentially abundant genera between container types, the percent abundances of each genus were compared between container types at each collection site. For Site 1 and 2, there were four differentially abundant genera between container types whereas Site 3 had eight (S9 Fig and S3 Table). Interestingly, we observed two genera (Acinetobacter and Allorhizobium-Neorhizobium-Pararhizobium-Rhizobium) with higher differential abundance in the plastic bucket container type from all three collection sites.

### Bacterial microbial abundance in artificial containers drives an increase in ZIKV infection rates

**Fig 6:**
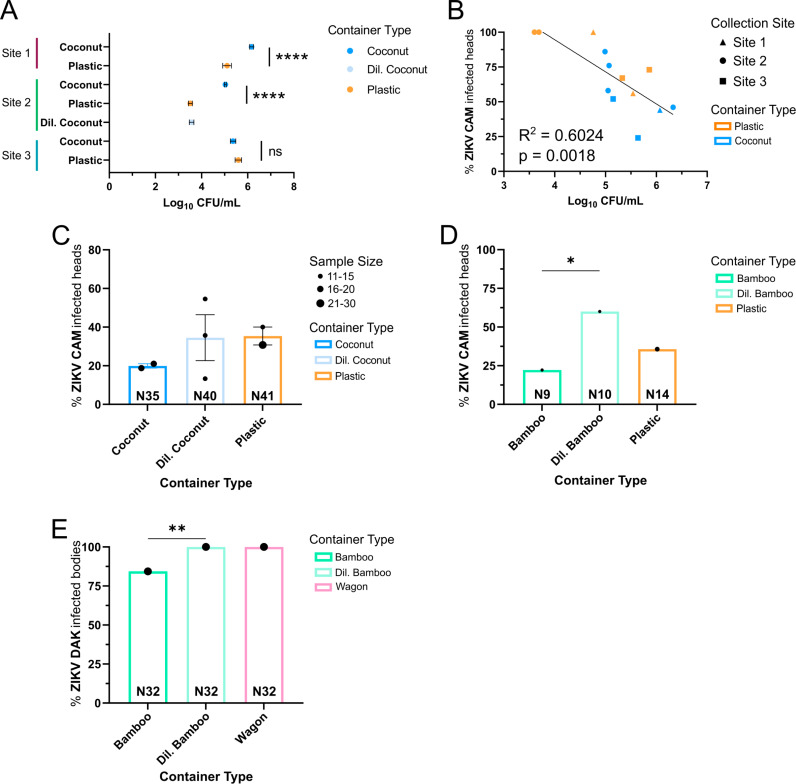
Abundance of bacteria is related to infection susceptibility. (A) Quantity of bacteria in the glycerol stocks used as a bacterial source in the gnotobiotic assay was cultured on LB agar and the number of colonies was counted. Graph represents the average log_10_ transformed CFU/ml from two independent experiments. Plotted by log_10_ CFU/ml on the X-axis. Error bars represent SEM. Statistical significance of the mean log_10_ CFU/ml was assessed by one-way ANOVA with Tukey’s multiple comparison tests. (Site 1 Plastic vs. Coconut: p-value = 0.0286; Site 2 Plastic vs. Coconut: p-value < 0.0001; Site 3 Plastic vs. Coconut: p-value = 0.8933; Site 2 Coconut vs. Dil. Coconut: p-value < 0.0001; Site 2 Plastic vs. Dil. Coconut: p-value = 0.9974). (B) A correlation plot showing the proportion of infected *Ae. aegypti* heads from Fig 3B (Y-axis) plotted with each group’s respective mean Log_10_ CFU/ml (X-axis) (correlation, p-value = 0.0019). The black line is the regression line, and the R^2^-value indicates the squared Pearson’s correlation coefficient statistic. (C) Bar graph showing infection prevalence in *Ae. aegypti* (PKT line) heads exposed to ZIKV Cambodia (FSS 1302) after larval development in either a coconut-, a diluted coconut-, or a plastic-derived microbiome from either Site 1 or Site 2. The heads were collected 14 days post-infectious bloodmeal and proportion infected was determined using a focus forming unit assay. The error bars represent SEM. Data represent up to three independent experiments as indicated by the black dots. The size of the black dot represents the number of individuals per independent experiment. No statistical significance was found by Chi-square analysis of a 2 X 2 contingency table (p-value = 0.3296). (D) Bar graphs showing infection prevalence in *Ae. aegypti* (PKT line) heads exposed to ZIKV Cambodia (FSS 1302) after larval development in either a bamboo-, a diluted bamboo-, or a plastic-derived microbiome from Site 1. The heads were collected 14 days post-infectious bloodmeal and proportion infected was determined using a focus forming unit assay. Data represent one experiment. Chi-square analysis of a 2 X 2 contingency table was performed as a function of infection (Bamboo vs. Diluted Bamboo: p-value = 0.0479; Dil. Bamboo vs. Plastic: p-value = 0.1196; Plastic vs. Bamboo: p-value = 0.2463). (E) Bar graphs showing the proportion of infected *Ae. aegypti* bodies (NGO line) exposed to ZIKV Dakar (41524) after larval development in either a wagon-, a bamboo-, or a diluted bamboo-derived microbiome collected from Site 1. Bodies were collected 14 days post-infectious bloodmeal and proportion infected was determined using a ZIKV-specific RT-PCR assay. Data represent one experiment. Chi-square analysis of a 2 X 2 contingency table was performed as a function of infection (Bamboo vs. Wagon: p-value = 0.0199; Dil. Bamboo vs. Wagon: p-value = 0.0199). (C-E) The total number of individuals for each group tested is displayed at the bottom of the bar.

Given that previous reports have documented that urban containers contain a higher bacterial load than sylvatic containers [17], we quantified the total bacterial abundance from each collection. To determine the initial amount of bacteria being introduced to the larvae, each preserved glycerol stock was serial diluted and plated on LB agar. Following incubation, the number of colonies was counted to quantify the total microbial abundance. The preserved coconut glycerol stocks had significantly higher bacterial abundance for Site 1 and Site 2 whereas Site 3 had similar abundances for both coconut- and plastic-derived glycerol stocks (Tukey’s multiple comparisons; Site 1 Coconut vs. Plastic: p-value < 0.0001; Site 2 Coconut vs. Plastic: p-value < 0.0001; Site 3 Coconut vs. Plastic: p-value = 0.7969) (Fig 6A). The bacterial burden varied over time but normalized across all flasks around day four or five (S4 Table). To confirm that inoculation of the gnotobiotic flasks with a lower abundance of bacteria results in fewer bacteria in the larvae, we inoculated with gnotobiotic flaks with a serially diluted complex bacterial community (bamboo shoot) and quantified the amount of bacteria. Larvae exposed to a reduced bacterial abundance retained fewer bacteria compared to larvae exposed to more abundant bacterial community (S10 Fig).

To assess the relationship between the bacterial abundance and infection rates across all collection sites and container types, the bacterial abundance (average Log_10_ CFU/ml) was plotted with its corresponding ZIKV infection rate. Correlation analysis demonstrated that higher infection rates correlated with a lower bacterial abundance (Pearson correlation coefficient (r) = -0.7761, p-value = 0.0018, R^2^ = 0.6024) (Fig 6B).

To directly test if the bacterial abundance observed in the different container types can drive variation in ZIKV infection rates, the bacterial concentration (CFU/ml) of the coconut-derived glycerol stock from Sites 1 and 2 was diluted (Fig 6A). Larvae were reared in coconut-, diluted coconut-, or plastic-derived microbiome prior to being challenged with ZIKV (Cambodia) as an adult (S1 Table). As previously observed, the individuals exposed to the plastic-derived microbiome were more susceptible to ZIKV infection than those exposed to the coconut-derived microbiome (Fig 6C). When exposed to the diluted coconut-derived microbiome, infection rates increased to levels similar to that of the plastic-derived microbiome exposure (Fig 6C). The differences in infection rate were not statistically significant when performing a chi-square analysis on 2 x 2 contingency tables, likely due to low sample sizes in the replicates and not enough statistical power. Additionally, the blood meal titer was lower in this experiment (mean blood meal titer: 5.4 log_10_ FFU/ml) resulting in lower infection rates overall, which could have affected our ability to determine statistical significance.

To demonstrate that the connection between ZIKV infection and total bacterial abundance during larval development is consistent across bacterial communities, we repeated the experiment with the bacterial communities preserved from a plastic container and bamboo shoot (August 2021 Site 1 collection, see materials and methods). These containers were the same used in used in S1 Fig (May 2021 Site 1 collection) which contained a different composition of bacterial taxa than the 2023 collections and was not dominated by *Vibrio spp*. (S11 Fig). Larvae were either exposed to the bamboo-, a 10-fold diluted bamboo-, or the plastic-derived microbiome and later challenged with ZIKV. Consistent with the diluted coconut-derived microbiome from the 2023 collections, larvae exposed to the diluted bamboo-derived microbiome also exhibited a high level of infection compared to larvae exposed to the bamboo-derived microbiome (Chi-square on 2 X 2 contingency table, p-value = 0.0479) (Fig 6D). To further test whether the bacterial abundance associated with the two container types was related to the differences in adult susceptibility to ZIKV, we repeated this experiment with microbiomes preserved from an additional artificial container (wagon) and natural container (bamboo) (June 2022 Site 1 collections, see materials and methods) and in an additional mosquito line (NGO) representing a different genetic background [31]. Quantification of total bacterial abundance indicated that the bamboo-derived microbiome had a higher bacterial load than the wagon-derived microbiome (S12 Fig). Larvae were either exposed to the bamboo-, a diluted bamboo-, or the wagon-derived microbiome and later challenged with a different isolate of ZIKV (DAK 41524) (S1 Table). The proportion of infected individuals exposed to the wagon-derived microbiome was higher than those exposed to the bamboo-derived microbiome (Chi-square on 2 X 2 contingency table, p-value = 0.0199) (Fig 6E). Consistent with the diluted coconut-derived microbiome, the diluted bamboo-derived microbiome also exhibited a high level of infection equivalent to the wagon-derived microbiome (Chi-square on 2 X 2 contingency table, p-value = 0.0199) (Fig 6E). Repetition of the experiment using independent microbiomes collected from different containers, a genetically diverse mosquito line, and infection with a different isolate of ZIKV provide confidence that exposure to a reduced bacterial load during development increases ZIKV infection rates.

## Discussion

Here, we expand on previous work demonstrating that larval exposure to different bacterial communities is important for arbovirus infection [15,24,25] and demonstrate that larval exposure to specific container-derived microbiomes drives differences in ZIKV susceptibility. We found that within a single environment (collection site) the proportion of ZIKV-infected bodies was dependent on exposure to microbiomes derived from a specific container type (bamboo shoot vs. plastic bucket). When larvae were exposed to microbiomes preserved from additional container types (coconut husk and plastic bucket) placed at multiple environments (collection sites), we observed that the proportion of ZIKV-infected heads was dependent on both the collection site and the container type, but across all collection sites ZIKV infection rates were higher in mosquitoes exposed to plastic-derived microbiomes during larval development. The bacterial community structure in the larvae differed between the plastic- and coconut-derived communities at each collection site, but a conserved plastic- or coconut- derived bacterial community across collection sites was not identified. However, we observed that the plastic-derived microbiome had fewer total bacteria than the coconut-derived microbiome and the proportion of ZIKV-infected individuals was dependent on the amount of initial bacteria introduced at the larval stage. Together, these data suggest that larval exposure to specific container type-derived microbiomes alters adult susceptibility to ZIKV, largely driven by differences in the amount of total bacteria between container types independent of bacterial composition.

The importance of the microbiome in arbovirus infection in the mosquito has been well documented [15,21,22,25,32–34]. Exposure of single isolates at both the adult stage [19,21,22,33] and larval stage [15,25] has been shown to alter the proportion of mosquitoes that become infected as well as the amount of replicating virus in the mosquito. Here we demonstrate that larval container-specific differences in bacterial communities can result in difference in ZIKV infection. We hypothesized that either differences in bacterial community structure (beta diversity), the complexity of the bacterial community (alpha diversity), presence of specific bacterial taxa, or the total bacterial abundance between container types could be driving the observed differences in ZIKV infection. Our data suggest that differences in bacterial abundance during larval development is responsible for the increased ZIKV infection we observed in the adults exposed to the plastic-derived microbiome. We observed differences in the total bacterial load between the microbiome preserved from plastic buckets compared to coconut husks. In two out of the three collection sites, the coconut-derived microbiome had more total bacteria that the plastic-derived microbiome. This was determined by counting the number of colony forming units using a culture-dependent assay. Given the small number of culturable bacteria, it is possible that we do not see a difference in total abundance of bacteria in Site 3 because we cannot culture the relevant bacterial species. Importantly, diluting the amount of bacteria in the coconut-derived or bamboo-derived microbiome to match the quantity of bacteria in the plastic-derived microbiome or wagon-derived, respectively, restored the infection rates to that of the plastic-derived or wagon-derived microbiome. Knowing that we identified different bacterial genera and different bacterial community structures between plastic-derived and coconut-derived microbiomes, this effect is independent of the bacterial species present or the bacterial community structure. This was observed across different containers (bamboo vs. coconut and different plastic containers (wagon vs plastic bucket)), mosquito genotypes, and viral genotypes. Previous studies have demonstrated the influence of the larval microbiome on ZIKV susceptibility is dependent on the mosquito genotype [25], but here we see the abundance of bacteria in the larval water influences ZIKV infection across mosquito genotypes.

These results are in accordance with a previous study that demonstrated that larvae reared in water seeded with a microbiome from environmental water (collected at a cemetery) produced less susceptible adults compared to laboratory tap water [24]. The bacterial abundance was shown to be higher at day 0 in the environmental water compared to the lab tap water [24]. In addition, this study by Louie et al. implicates the complexity (how many different bacterial species are present), or alpha diversity, of the larval microbiome was capable of influencing ZIKV infection outcomes in adult mosquitoes [24]. Although we could not detect any differences in alpha diversity between our plastic- and coconut-derived communities that were consistent across environments, we did observe that the bamboo-derived community had a higher alpha diversity than the plastic-derived community in a single environment and urban sites have been shown to have a lower alpha diversity compared to sylvatic sites in the field [15]. Although this study implicates the total abundance of bacteria in the plastic-derived community in influencing ZIKV infection rates, it is possible that differences in alpha diversity between bacterial communities also contribute. In fact, the contribution of these two variables is difficult to disentangle given that dilution of the complex community will likely dilute out taxa present at low abundance and could simplify the bacterial diversity and change the alpha diversity of the sample.

Given previous work showing that exposure to different bacterial isolates and/or communities drives variation in adult susceptibility to arboviruses [15,24,25], we initially hypothesized that our observed differences in ZIKV infection rates could be explained by conserved plastic- or coconut-specific bacterial communities in the larvae that were consistent across collection sites. In other words, we initially expected to see bacterial communities derived from plastic containers to cluster independently from coconut containers regardless of collection site, similar to what was observed in the inoculation water. But instead, we observed that the initial bacterial community structure of water sources clustered by container type, independent of the collection site, but once the microbial community colonized the larvae, the bacterial community structure between container types was no longer shared across each collection site. These data suggest that the bacterial communities in the container water have predictable differences between a container type across multiple environments, but once they colonize the larvae the bacterial community structure within the mosquito is dependent on both the collection site and container type, likely a result of competition between bacteria in the mosquito gut-driven by differences in the availability of bacterial species in each environment [35,36]. This is not surprising given that the larval microbiome is largely determined by the environment [11,15,37–43]. This data suggests that something beyond differences in bacteria community structure between environments has meaningful differences in the adult interactions with human pathogens.

In addition to bacterial community structure, we compared the abundance of specific bacterial genera between larvae exposed to the plastic-derived versus coconut-derived microbiomes. Two bacterial genera were higher in the plastic-reared larvae compared to coconut-reared larvae across all three collection sites, Acinetobacter and *Allorhizobium-Neorhizobium-Pararhizobium-Rhizobium* (ANPR). These genera were detected in the coconut-derived microbiomes but at lower abundances. Both these genera are commonly identified members of the mosquito microbiome [44–47]. It is possible that the abundance of either *Acinetobacter* or ANPR could be influencing the differences in infection rates, however, further work isolating these genera and supplementing them into the gnotobiotic system to investigate their potential to alter arbovirus susceptibility remains to be done. Our data demonstrating that we can dilute the bacterial communities from coconut (where these genera were identified as significantly reduced compared to plastic-derived communities) as well as bamboo and increase infection to the level of the plastic exposed provides confidence that the difference we see in infection rates between plastic and coconut exposed is not a result of the presence of these two genera.

While we observed that the larval samples exposed to the plastic bucket- or coconut-derived communities were largely populated by OTUs mapping to *Vibrio* spp., it is unlikely that the correlation between ZIKV infection rates and bacterial abundance is a Vibrio-specific effect, but instead a community level effect. We were not able to culture a *Vibrio spp*. from these samples to directly test the influence of *Vibrio spp*. abundance compared to other taxa, but our observation that diluting coconut- or bamboo-derived communities originating from multiple different containers over different time periods increases ZIKV infection compared to the undiluted samples provides evidence this is a community level effect.

Another alternative hypothesis to explain the increased ZIKV infection in those exposed to the plastic-derived bacteria during larval development is that larval exposure to different bacterial communities results in different bacterial taxa that can be transstadially transmitted or it has facilitated the proliferation of specific bacterial taxa in adults and these adult microbial communities are influencing ZIKV infection. Indeed, a possible carry-over effect of larval exposure to different bacterial communities is changes to the adult microbiome, which itself could be influencing ZIKV infection. To fully address this, characterization of the bacterial communities in the adults following larval exposure to different bacterial communities would need to be performed. Additionally, if different taxa are identified they would need to isolated and evaluated in a functional assay to assess their contribution to ZIKV infection. Here we only focus on characterizing the larval microbiome, but this represents a future direction that would provide further insight into how larval exposure to different bacterial communities results in changes in adult interactions with arboviruses.

Gut microbes are important for the nutritional health of mosquitos [48–50] and interact with metabolic processes [51–53]. Variations in the nutrition status at the larval stage can alter adult size and development [54]. Furthermore, adult size and fitness can influence mosquito infection rates [55,56]. Bacteria in the larval water are a source of nutrition, and the amount of food larvae have is important [49,57–59]. Furthermore, metabolic changes, specifically lipid metabolism, arising through exposure to different bacterial species at the larval stage can carry over to the adult [51]. Lipid metabolism plays a critical role in the replication and dissemination of arboviruses in mosquitoes [60–65]. Given the importance of larval nutrition and metabolism on adult interactions with arboviruses, it is possible that the differences we observed in ZIKV infection rates are a result of perturbation of the mosquito metabolism arising from different amounts of bacteria (aka food) present in the larval development water.

The use of preserved microbial communities as a source of the microbiome for larvae in the gnotobiotic system has been documented before. Previous work has used preserved complex microbiomes created from homogenized larvae [23] as well as pelleted microbes from environmental water [24]. Additionally, a recent study demonstrated that the cryopreservation of bacteria in glycerol for use in the gnotobiotic system had little effect on the microbiome acquired by gnotobiotic larvae meaning that glycerol stocks are an effective way to preserve and store reproducible bacterial sources [26,27]. While not able to recapitulate the field microbiome exactly, this represents a meaningful tool to study the influence of field microbiomes.

Given the high environmental variation of microbial species in the field that mosquitoes are exposed to [66] and knowing that the microbiome of mosquitoes is largely determined by the environment [11,15,37–44], it is challenging to extend these results to a natural setting.

While it is tempting to extend the results of this study to field relevance, further work needs to be done to understand if *Ae. aegypti* development in urban containers produces more susceptible adults than development in sylvatic containers. Here, we only tested the influence of one representative urban breeding site, plastic buckets. In reality, *Ae. aegypti* is ovipositing in a multitude of urban containers in the field including large drums, tires, and tin cans, etc. All of which may shape the bacterial communities in the water differently, but characteristics such as bacterial abundance and species diversity are likely conserved between urban and sylvatic sites [15]. Characterization of oviposition sites in Rabai, Kenya demonstrated that urban sites have lower bacterial density than those found in the sylvatic [17] which suggests that the differences in bacterial abundance we observed in the mock oviposition sites reflect the conditions found in the field. Our use of multiple representative sylvatic containers (bamboo shoots and coconut husks) provides confidence that these results may extend to the field. Oviposition sites in the field are subjected to environmental fluctuations including changes in the water level and temperature. Moreover, other organisms such as other insects can be present leading to resource competition. The gnotobiotic system is limited and not a direct correlate for the larval-container interactions occurring in the field, but it is a unique tool that allows us to investigate broad microbiome characteristics. Recapitulating the entire characteristics of a field larval container is not relevant to this study. Here, we aimed to isolate the influence of the microbiome.

Overall, our observation that microbiomes from a specific container type across multiple environments result in predictable changes in adult ZIKV infection rates paves the way for future in-depth field studies.

## Materials and methods

### Field sampling

Water from mock larval breeding sites was collected in Texas in May 2021, August 2021, June 2022, and May 2023. The 2021 collections were done using bamboo shoots and a plastic bin located at Site 1 (Fig 2A) with approximately 5 meters between the containers. These containers had been present in the environment for months to year as the bamboo was naturally growing at Site 1 and the plastic bucket was found outside. The 2022 collection was also done at Site 1 (Fig 2A) with bamboo shoots and a small plastic wagon with approximately 5 meters between the containers. Similar to the 2021 collections, the containers in the 2022 collection had been present in the environment for an extended period of time. The 2023 collection was done using halved coconut husks (two halves per site, pooled when collected) and large empty plastic containers (plastic bucket, one per site) purchased at the same time for each site (Fig 2B) with at least 5 meters between the containers. Prior to transfer to the field, containers were either cleaned with dish soap and water (plastic containers) or rinsed with water (coconut husks). Containers were left outside for seven days (except for the bamboo shoots which were permanent) during which there was significant rainfall on multiple days. Following a heavy rainstorm (day six), water was collected and transported to the lab (day seven). Within a laminar flow cabinet, water was mixed with glycerol (final concentration of 25%) and aliquoted into conical tubes (50ml or 15ml) which were frozen at -80°C. For the 2023 collection, unaltered water (400µl) was spotted on a Whatman FTA card, allowed to dry, and stored at room temp. Additional information on each collection site can be found in S2 Table. Water was not filtered prior to freezing to isolate the bacteria therefore other microbes may be present in the preserved glycerol stocks such as fungi.

### Gnotobiotic larvae

Axenic larvae were generated using the generations 9–14 of *Ae. aegypti* laboratory colonies derived from a natural population collected from Kedougou (KED), PK10 (PKT), or Ngoye (NGO), Senegal [31]. The KED and PKT lines originate from the southeastern region of Senegal. Kedougou is a city on the edge of the PK10 forest and the natural population oviposits in both natural and artificial containers. The PKT line originates from a sylvatic population that exclusively oviposits in natural containers. The initial study was conducted with the KED line but due to rearing issues, we switched to the PKT line for further studies since both colonies originated from the same region. Ngoye is an urban city in the Northwest region of Senegal where the natural population oviposits in artificial containers. Whole genome sequencing has confirmed that NGO is genetically distinct from KED and PKT lines [31]. Eggs were gently scraped off the egg paper into to a 50ml conical tube. The eggs were soaked in 70% ethanol for 5 min, 3% bleach for 3 min, and 70% ethanol for 5 min. During the final ethanol wash, eggs were transferred to a new conical tube with a filter top before being rinsed in sterile water three times and then allowed to hatch in 30ml of sterile water using a vacuum chamber. After hatching, the larvae were transferred to sterile T150 cell culture flasks with filter-top lids containing 125ml of sterile water. Larval density was 100–150 per flask. The larvae were provided 150µl of sterile fish food initially and then 300µl every day (day 2 post-hatching onwards). The water within the larval flasks was not changed for the duration of the experiment. Sterile fish food was produced by grinding fish food (TetraMin Tropical Flakes, Tetra, Melle, Germany) with a mortar and pestle and resuspending it in water (10g/ml) and autoclaving it for 30 min. To create gnotobiotic larvae, 3ml of a thawed glycerol stock of water collected from one of the containers placed at each collection site was added to a flask of axenic larvae. Larval flasks were stored under standard insectary conditions (28°C, 12-hour light/12-hour dark cycle and 70–80% relative humidity). For each experiment, two or three L3/L4 larvae were collected from replicate flasks. Larvae were frozen at -20°C for DNA extraction. To ensure sterility of the axenic larvae, a control flask (25ml) with 10–20 larvae was maintained for the duration of each experiment and provided food and none of them developed past L1 as previously described for axenic larvae [11]. Pupae were collected from the flasks in a laminar flow cabinet to prevent the introduction of environmental microorganisms to the larvae. Pupae were rinsed in deionized water and transferred to cups or cages (BugDorm-4E2222 Insect Rearing Cage, BugDorm, Taiwan) to emerge with free access to a 10% sucrose solution. Following pupation and eclosion, adults were maintained under standard insectary conditions and allowed to be colonized by environmental bacteria. This was done because we were measuring the carry-over effects of the larval microbiome and wanted the adult microbiome to be seeded under standard insectary conditions.

### Pupation rate

Pupae were counted from the onset of pupation (Day 5) until approximately 80–90% of the larvae pupated (Day 10) in the same flasks (three or five) used for the adult viral challenge assays. Larvae that did not pupate were counted and considered in the total amount of individuals. To determine the rate of pupation, the percent pupae was determined at each day by dividing the number of pupae by the total number of individuals. Data from two independent experiments was used, each with three or five internal replicates (three or five replicate flasks, in one experiment a flask was lost for Site 1 Plastic reducing the number of flasks to two). GraphPad Version 9 (GraphPad, San Diego, CA) was used to generate a simple logistic regression that computed the day that 50% of larvae had pupated (PD_50_). An ANOVA was run on the summary statistics of the PD_50_ generated from the logistic regression to determine if the PD_50_ was dependent on the bacterial source, the collection site, or an interaction between the two (S3 Table). One-way ANOVA was performed with Tukey’s multiple comparison tests to compare the mean PD_50_ between groups. Data are a summary of two biological experiments done with three or five replicate flasks each time. The number of larvae used per experiment along with statistical information associated with each comparison are listed in S3 Table.

### DNA extractions

#### Water/glycerol stocks.

Water samples across multiple stages of the experimental setup were included to ensure consistency. After the water was collected and returned to the lab, 400µl of each sample was added to two discs on a Whatman FTA card (Whatman, Maidstone, UK) under a laminar flow hood and stored at room temperature until further processing. In addition, a sample of water (1.5ml) without glycerol was collected under a laminar flow hood and frozen at -80ºC until further processing. For the preserved glycerol stocks, leftover volume from experimental setup was used.

#### Whatman FTA card – unfrozen water.

Extraction was done by following the organic extraction of DNA from Whatman [67]. Briefly, sterilized scissors were used to cut out the two discs and both were placed into a sterile 2ml tube. To ensure the paper was covered, 1ml of extraction buffer (10 mM Tris-HCl, pH 8.0; 10 mM EDTA, disodium salt, pH 8.0; 100 mM sodium chloride; and 2% v/v SDS) and 40µl of Proteinase K stock solution (20 mg/ml in sterile distilled water) was added to each sample. After briefly vortexing, samples were placed in a shaking incubator at 56°C with 300rpm overnight. Samples were transferred to a new tube to prevent overflow of volume and 1ml of buffered phenol (pH 8.0) was added to the new tube before vortexing and centrifuging at max speed (21000xg) for 10 minutes. The upper aqueous phase was transferred to a new tube containing 500µl chloroform and centrifuged again at max speed for 10 minutes. The new upper aqueous phase of the samples was moved to a new tube containing 50µl of 3M sodium acetate (pH 5.2) before 800µl of 100% ethanol was added. After mixing, the samples were allowed to precipitate at -20°C for at least 1.5h. Samples were centrifuged at max speed for 1h to pellet the DNA. Once the supernatant was removed, the pellet was washed with 70% ethanol (1ml) and centrifuged for 20 min at max speed. Ethanol was removed and pellets were allowed to air dry in a fume hood until ethanol had evaporated. Pellets were resuspended in 20µl of nuclease-free water.

#### Water frozen without glycerol.

Water samples were thawed at room temperature before centrifugation at max speed (21000xg) at 4ºC for 30 mins. Following the spin, 1ml of water volume was removed and discarded (leaving 500ml) and 200µl of sterile water was added to each sample. The negative control contained only 700µl of sterile water. After a brief vortex, 500µl of Buffer ATL (QIAGEN, Hilden, Germany) and 50µl of proteinase K (QIAGEN, Hilden, Germany) was added to each sample. Once vortexed to mix, the samples were placed in a shaking incubator at 56ºC with 300rpm overnight. On the following day, 200µl of Buffer AL (QIAGEN, Hilden, Germany) and 200µl of ethanol were added to each sample and vortexed before continuing with the remainder of the protocol for the QIAmp DNA Extraction kit (QIAGEN, Hilden, Germany) by following the manufacturer’s protocol. Samples were eluted in 50µl of nuclease-free water.

#### Water frozen with glycerol (Glycerol stocks).

Following setup of a gnotobiotic experiment, 1ml of the remaining glycerol stock was transferred to 2ml Eppendorf tubes. The samples were centrifuged at max speed (21000xg) at 4ºC for 1.5h. Most samples lacked a visible pellet and after discarding the supernatant, approximately 20µl of each sample was left in the tube. ATL buffer (500µl) and proteinase K (50µl) was added to each sample to resuspend the pellet. The samples were placed in a shaking incubator at 37ºC with 200rpm overnight. On the following day, 200µl of AL buffer was added and samples were incubated at 70ºC for 10 mins. After briefly vortexing 200µl of ethanol was added to each sample add vortexed again. The extraction was finished by following the manufacturer’s protocol for the QIAmp DNA Extraction kit (QIAGEN, Hilden, Germany).

#### Gnotobiotic larvae (L3/L4).

Prior to extraction, gnotobiotic L3/L4 larvae were surface sterilized by soaking in 70% ethanol for 10 min and rinsing three times in sterile water. DNA from gnotobiotic larvae were extracted individually following a previously described assay [15] using the QIAmp DNA Extraction kit (QIAGEN, Hilden, Germany). Briefly, individuals were added to a sterile tube with 1mm glass grinding beads (Research Products International Corp) containing 300μl of lysozyme (20mg/ml) dissolved in ATL buffer. The samples were homogenized for 3 rounds of 20 secs with a 30 second pause between every round (Precellys Evolution, Bertin Technologies) then incubated at 37°C for 2 hours. Following incubation, 20µl of proteinase K (Invitrogen) was added to each sample and briefly vortexed after which the samples were incubated at 56°C for 4 hours on a shaker (300rpm). After the second incubation, 200µl of Qiagen AL buffer and 200µl of 100% ethanol were added to the samples and briefly vortexed. Extraction was continued following the manufacturer’s protocol and eluted in 20µl of nuclease-free water. To control for contamination of bacteria introduced during the DNA extraction, a blank sample was included on each day of extractions.

### 16*S* sequencing and metagenomic analysis

For the 2021 collections, libraries were made from DNA extractions from seven individual larvae from each treatment. For the 2023 collections, libraries were made from 3 plastic water samples (one of each extraction method), 3 coconut water samples, and 12–18 individual L3/L4 larvae resulting in a total of 110 samples. Additionally, a blank sample which consisted of the same sterile water that was used to wash the larvae in the surface sterilization was extracted with each batch of samples. The larvae from each collection site and container type were randomized across extraction batches (6 total batches), and a blank extraction was performed alongside each of the 6 extraction batches. For the 2021 collections, sequencing libraires were made using the Illumina 16S Metagenomic Sequencing Library Preparation protocol following the manufacturer’s protocol. DNA concentrations from each individual larvae were determined by Qubit and equal amounts of DNA from each individual library were pooled to generate the final library. To generate sequencing libraries for the 2023 collections each isolate were generated using the Zymobiotics kit (Zymo Research, Irvine, CA) following the manufacturer’s protocol. Equal amounts of qPCR amplicons were pooled to generate the final library. The pooled libraries were diluted to 4 pM and run on the Illumina Miseq using a MiSeq Reagent Kit v2 (500-cycles).

To identify known bacteria, sequences were analyzed using the CLC Genomics Workbench 21.0.5 Microbial Genomics Module (CLC MGM). Reads containing nucleotides below the quality threshold of 0.05 (using the modified Richard Mott algorithm) and those with two or more unknown nucleotides or sequencing adapters were trimmed out. Reference-based Operational Taxonomic Unit (OTU) picking was performed using the SILVA SSU v132 97% database [68]. Sequences present in more than one copy but not clustered to the database were placed into de novo OTUs (97% similarity) and aligned against the reference database with an 80% similarity threshold to assign the “closest” taxonomical name where possible. Chimeras were removed from the dataset if the absolute crossover cost was three using a k-mer size of six. OTUs with a combined abundance of less than two were removed from the analysis. Low abundance OTUs were removed from the analysis if their combined abundance was below 10 or 0.1% of reads. The number of reads per sample used in the analysis ranged from 5,364 – 42,083. Only reads that mapped to bacteria were kept. Taxa classified as “Ambiguous Taxa” are reads mapping to bacterial DNA, but that cannot be identified at the taxonomic level. OTUs from the negative controls overlapped with the samples and all OTUs mapping to the negative controls were removed from the analysis (S12 Fig).

Abundance profiling was performed using MicrobiomeAnalyst [69,70]. The analysis parameters were set so that OTUs had to have a count of at least 10 in 20% of the samples and above 10% inter-quantile range. Analysis was performed using actual and total sum scale abundances. Alpha diversity was measured using the observed features to identify the community richness using Chao1. Statistical significance was calculated using T-test/ANOVA. Beta diversity was calculated using the Bray-Curtis dissimilarity measure (genus level). Permutational Multivariate Analysis of Variance (PERMANOVA) analysis was used to measure effect size and significance on beta diversity for grouping variables [71]. Relative abundance analysis was done in MicrobiomeAnalyst at the level of genera.

Pairwise differential abundance of specific genera was done in MicrobiomeAnalyst. Statistical significance between groups was determined by EdgeR. Genera with a greater than or less than 1 log2 fold change and a FDR corrected p-value (Benjamin-Hochberg correction test) less than 0.05 were included in the final output. (S3 Table).

A total of 1836 OTUs were identified in the 91 individual larvae sequenced. After filtering, 65 OTUs remain which represent 14 genera. Sequences from three individuals were removed from the analysis because they did not achieve enough reads, one from Site 1 Coconut, Site 2 Coconut, and Site 2 Plastic. These final 65 OTUs were used for the analysis in the MicrobiomeAnalyst.

Out of the 18 water samples sequenced, two were discarded due to poor sequencing quality (Site 1 Coconut, flask and glycerol). A total of 1809 OTUs were identified in the remaining samples. After filtering, 161 OTUs remain which represent 38 genera. Sequences from one sample, Site 2 Plastic (Whatman) was removed because it did not achieve enough reads. These final 161 OTUs were used for the analysis in the MicrobiomeAnalyst.

### Gnotobiotic *Ae. aegypti* arboviral infection and focus forming assay

#### Artificial infectious blood meals.

Mosquitoes were orally challenged with the wild-type Zika virus Dakar (41524) or Cambodia (FSS 13025) isolate received from the World Reference Center for Emerging Viruses and Arboviruses (WRCEVA) at UTMB. Infectious blood meals were prepared as previously [25]. Briefly, if necessary, virus stock was diluted in cell culture media Dulbecco’s modified Eagle Medium (DMEM) (Gibco, Waltham, MA) with 1.5% heat-inactivated fetal bovine serum (FBS), and 1% penicillin/streptomycin (Pen-Strep, 100X)). Prior to addition to the blood, sodium bicarbonate (7.5%) was mixed with the virus stock at 1% final concentration. One volume of virus suspension was mixed with two volumes of defibrinated sheep blood (Colorado Veterinary Product) washed three times in 1X PBS. The blood meal was supplemented with 10 mM adenosine triphosphate (ATP). Females were offered an artificial blood meal for 15 minutes using the Hemotek system with de-salted pig intestine as the membrane. An aliquot of the bloodmeal was preserved to back-titer the blood meals to determine the actual infectious dose. See S1 Table for averaged titers. The titers for independent experiments can be found in the raw data file (S5 Table).

#### Focus forming assay.

To determine the infection status of the mosquitoes, a focus-forming assay was performed on the heads. Heads were frozen at -80°C prior to assay without media. Individual heads were placed into vials containing 0.1 mm glass beads and 200µl of supplemented cell culture media (DMEM with 1% Pen-Strep, 1% antibiotic and antimycotic (Gibco, Waltham, MA), 1.5% FBS) and homogenized for 3 rounds of 20 secs with a 30 second pause between every round (Precellys Evolution, Bertin Technologies, Montigny-le-Bretonneux, France). 24-well plates were seeded with Vero cells and incubated to reach confluency. Each well was inoculated with 200µl of head homogenate in 10-fold dilutions (from 10^-1^ to 10^-4^) in duplicate and incubated at 37°C (5% CO^2^) for 1 hour, rocking every 15 minutes. Infected cells were overlaid with Opti-MEM media (Gibco, Waltham, MA) supplemented with 1.25% carboxymethyl cellulose, 5% FBS, and 1% Pen-Strep. After three days of incubation at 37°C, infected cells were fixed with 10% formalin for at least 1 hour then washed three times in 1X PBS. Approximately 500µl of blocking solution (5% w/v non-fat powdered milk in 1X PBS) was added to each well and plates were rocked for 30 minutes. The blocking solution was discarded and 200µl of primary antibody (obtained from the WRCEVA at UTMB) solution diluted 1:1000 in blocking solution was added to each well and plates were placed on plate rocker overnight. The primary antibody solution was discarded, and plates were washed three times with 1X PBS prior to the addition of 200µl of secondary antibody (peroxidase-labeled goat anti-mouse IgG human serum, KPL-474–1806, SeraCare, Milford, MA) solution diluted 1:2000 in blocking solution. Plates were placed on the plate rocker for 1 hour. The secondary antibody solution was discarded, and plates were washed three times with 1X PBS. To develop visible foci, 100µl of TrueBlue peroxidase substrate (KPL 5510–0050, SeraCare, Milford, MA) was added to each well, and plates were placed on the plate rocker until foci could be seen, around 10 min. Plates were washed with deionized water and FFU was counted with the help of a light. Focus-forming units were log_10_ transformed to represent the concentration of infectious ZIKV particles detected in *Ae. aegypti* heads. Presence or absence of foci was used to determine infection status. Statistical analysis of the titers was done in GraphPad Version 9 using Two-way ANOVA and Tukey’s multiple comparisons using the following predictor variables: container type, collection site, and the interaction between them. Proportion infection was assessed using R (glm by infection~container*site and anova using the Chisq test).

#### ZIKV RT-PCR.

To determine the infection rate in the body (Figs 1 and 6D), whole female bodies were homogenized in 200 μl of a crude RNA extraction buffer (10 mM Tris HCl, 50 mM NaCl, 1.25 mM EDTA, fresh 0.35 g/L proteinase K (Invitrogen)) during two rounds of 3 minutes at a 30Hz/s frequency in a TissueLyser II grinder (QIAGEN, Hilden, Germany). Total RNA was converted into complementary DNA (cDNA) using M-MLV reverse transcriptase (Invitrogen) and random hexamers (Invitrogen), the reaction was carried out as follows: 10 min at 25°C, 50 min at 37°C, and 15 min 70°C. The cDNA (2.5µl) was amplified by PCR carried out in a 25μl reaction containing 12.5μl of 2X DreamTaq DNA polymerase (Thermo Fisher Scientific, Waltham, MA) and 10 μM of each ZIKV primer (forward: 5’-GTATGGAATGGAGATAAGGCCCA-3’, and reverse: 5’-ACCAGCACTGCCATTGATGTGC-3’). Cycling conditions were as follow, 3 min at 95°C, followed by 40 cycles of 30s at 95°C, 30s at 61°C, and 15s at 72°C with a final extension step of 5 min at 72°C. Amplicons were visualized on a 2% agarose gel. The proportion of ZIKV-infected females was analyzed by Chi-square analysis on a binomial logistic regression as a function of container, and collection site in R.

### Bacterial quantification - Culture-based assay

The amount of bacteria in the glycerol stocks used to seed the gnotobiotic larvae or the flasks was quantified with a culture-based assay. Thawed glycerol stocks or flask water were serially 10-fold diluted (-1 to -6 or lower if needed) and plated using the drop method onto LB agar plates (glycerol stocks were generally neat to -5). Briefly, 30µl was taken from a stock or a flask (1ml was moved into a 1.5ml tube to prevent contamination of the flask) and serially diluted across a 96-well cell culture plate. Glycerol stocks were serially diluted once and plated five to six times per sample. Flask water was done in duplicate for each flask and plated once per serial dilution. Using a 10µl micropipette, 10µl of the dilutions was then carefully added to a pre-labeled agar plate as droplets to create a grid pattern (either 6 x 6 or 5 x 6). Plates were incubated for 36–48 hours (overnight for flask water) at 30ºC before the number of colonies was counted and used to calculate colony forming units (CFU)/ml. The total count of CFU from at least two drops at the countable dilutions were determined and averaged. Experiment was conducted twice for each glycerol stock except for Site 2 which was repeated a total of four times due to the dilution experiment. Data were analyzed with GraphPad Prism (Version 9) one-way ANOVA (p-value <0.0001) with Tukey’s multiple comparison tests (Site 1 - Plastic vs. Coconut: p-value = 0.0286; Site 2 - Plastic vs. Coconut: p-value < 0.0001; Site 3 - Plastic vs. Coconut: p-value = 0.8933; Site 2 - Coconut vs. Dil. Coconut: p-value < 0.0001; Site 2 - Plastic vs. Dil. Coconut: p-value = 0.9974; additional p-values can be found in S2 Table). Flask data is from the same flasks (three or five) used for the adult viral challenge assays. One experiment includes days 0, 2, 4, 6, and 8 whereas the second experiment includes days 0, 2, 5, and 6 (S3 Table).

### Bacterial quantification - qPCR assay

DNA was extracted from glycerol stocks as described above for the water samples collected from a wagon and bamboo shoot (June 2022). To establish a quantitative standard curve, DNA extracted from E. coli was amplified using the PCR primers 331F/797R [72] and gel purified with a QIAquick Gel Extraction Kit (QIAGEN, Hilden, Germany). A DNA copy number calculator [73] (Thermo Fisher Scientific, Waltham, MA) was used to determine the amount of copies based on the concentration of DNA. qPCR reactions were performed in duplicate using GoTaq qPCR Master Mix (2X) (Promega, Madison, WI) in a 20µl reaction containing 10µl of 2X qPCR Master Mix, 2µl of 4uM primer (331F/797R) and 1–2µl of DNA, with the remaining volume made up of nuclease-free water. Cycling conditions were as follows: 2 min at 95°C, 40 cycles of 15s at 95°C, and 1 min at 55°C, followed by a melt curve from 60ºC to 95ºC (15s, 0.5ºC/s). The copy number determined by the calculator was plotted against the mean Ct value for the standard curve (serial dilution of the E. coli DNA) and the equation used to calculate the copy number for the unknown samples. Experiment was done twice with both 2µl and 1µl of DNA to ensure values were consistent. All reactions were performed on a QuantStudio 6 Real-Time PCR System (Applied Biosystem, Foster City, CA).

### Quantification of bacterial abundance in larvae

Following the gnotobiotic assay as described above, larval flasks were seeded with the Bamboo Shoot glycerol stock (June 2022) undiluted, a 1:10 dilution or a 1:100 dilution of the same stock (one flask per dilution). Larvae were collected on Day 2 and surface sterilized as described above. Three pools were made of three larvae, each measuring approximately 2–3 mm and designated as L2 [74]. Homogenization was done by hand with a disposable micro-pestle for approximately 15 seconds in a 1.5ml tube with 100µl of sterile nuclease-free water. Samples were serially diluted and 50µl was plated in duplicate (-1 to -4) on LB agar plates and incubated overnight at 30°C. Countable dilutions were used to calculate CFU/ml. Data were analyzed with GraphPad Prism (Version 9) one-way ANOVA (p-value = 0.0015) with Tukey’s multiple comparison tests (Undiluted vs. 1:10: p-value = 0.0416; Undiluted vs. 1:100: p-value = 0.0012; 1:10 vs. 1:100: p-value = 0.0258).

### Microbiome dilution assays

Following the gnotobiotic assay as described above, larval flasks were seeded with either the coconut or plastic glycerol stock (May 2023, Fig 6C) or a diluted coconut glycerol stock from Sites 1 and 2 in up to three experimental replicates. The diluted coconut stock was diluted by added sterile water to achieve the mean bacteria CFU/ml of the plastic glycerol stock. Given repeated issues with feeding rates of the mosquitoes following gnotobiotic treatment, only groups with at least 10 individuals were kept for analysis. For Fig 6D, either the bamboo or plastic glycerol stock were used (August 2021) and the diluted bamboo was created by a 1:10 dilution of the bamboo using sterile water. For Fig 6E, either the bamboo or wagon glycerol stock were used (June 2022) and the diluted bamboo was diluted to the Log_10_ 16S gene copy number per µl of the wagon by adding sterile water.

### Statistical analysis

Statistical analysis was conducted using GraphPad Prism software version 9 and R v4.4.1 (www.r-project.org). All statistical output can be found in S3 Table. All raw data can be found in S5 Table.

## Supporting information

S1 Fig Bacterial community structure differs between gnotobiotic larvae exposed to container-derived water. The structure of bacterial communities was determined by deep-sequencing the V3-V4 region of the bacterial 16S gene in individual larvae reared in water preserved from a bamboo shoot or a plastic bucket in a gnotobiotic system. **(A)** Alpha diversity. Boxplot of the Chao1 diversity index to determine differences in bacterial diversity between samples (Welch t-test, p < 0.001). **(B)** Beta diversity. Principal component analysis of Bray-Curtis dissimilarity index based on genera abundance to determine similarities between the community structures of the sample types (PERMANOVA, p-value = 0.003). Data represents 1 independent replicate. The number of individual larvae represented is Bamboo: 7; Plastic: 7.
(EPS)

S2 Fig Consistent pupation rates between larvae reared in different container-derived microbiomes. Variation in pupation rate are shown for *Ae. aegypti* reared in the presence of bacteria from a container-associated preserved glycerol stock in a gnotobiotic system. Graph represents the average day on which 50% of the larvae had pupated (PD_50_) for each site and container type determined using a simple logistic regression for each flask. Pupation rate was determined by daily pupae counts in three or five flasks (in one experiment a flask was lost for Site 1 Plastic reducing the number of flasks to two) of gnotobiotic larvae in two independent experiments. Flasks contained an average of 159 larvae. Axenic larvae included in each independent experiment failed to pupate. Statistical significance of the PD_50_ was assessed by one-way ANOVA (p-value = 0.9108) with Tukey’s multiple comparison tests (p-values can be found in S2 Table). Error bars represent SEM.
(TIF)

S3 Fig Larval development in container-derived microbiomes does not alter disseminated viral titer in a site or container specific way. Boxplot showing the dissemination titers of infectious ZIKV particles expressed as the Log_10_-transformed number of focus-forming units (FFU) per ml detected in the *Ae. aegypti* head fourteen days post-infectious blood meal (Fig 3, Dose III). Each point represents an individual and the mean is represented by a horizontal line. The error bars represent the min and max. Data represent two independent experiments expect for Site 1 coconut which has one. Data were analyzed by Two-way ANOVA as a function of container type, collection site, and their interaction (container type: p-value = 0.2503, collection site: p-value = 0.8849, container type x collection site: p-value = 0.0160). The number of positive individual mosquitoes represented is Site 1 Coconut: 18; Site 1 Plastic: 32; Site 2 Coconut: 38; Site 2 Plastic: 37; Site 3 Coconut: 16; Site 3 Plastic: 19.
(TIF)

S4 Fig Bacterial community structure differs between types of container-derived water. The structure of bacterial communities was determined by deep-sequencing the V3-V4 region of the bacterial 16S gene in water collected from a coconut husk or a plastic bucket. Each point represents one extraction method. Coconut Site 1 had poor sequencing quality for two of the three samples so only the Whatman paper is included in the analysis. Additionally, Plastic Site 2 (Whatman) had did not achieve enough reads and was removed. Principal component analysis of Bray-Curtis dissimilarity index based on genera abundance was used to determine similarities between the community structures of the samples (PERMANOVA, p = 0.001).
(EPS)

S5 Fig Rarefaction curves showing the sequencing depth of each library. The number of species is shown on the Y axis, and the number of sequencing reads is shown on the X axis.
(EPS)

S6 Fig Bacterial community diversity is similar between gnotobiotic larvae reared in plastic and coconut water sources. Alpha diversity index (Chao1 diversity index) was represented as box plots for each container type/collection site. The median was defined as the line inside each box while the interquartile range (IQR) between the 25th and 75th percentile was delimited by the outer box. The y-axis represents the Chao1 index. ANOVA analysis yielded a p-value = 0.26344.
(EPS)

S7 Fig Community structure differs between container type within collection sites and between container type independent of collection site. **(A-C)** Beta diversity metrics for each collection site analyzed separately by container type and **(D-E)** by container type across all three collection sites. The dissimilarities between **(A-C)** the two container types (coconut and plastic) in each of the three collection sites and **(D-E)** the container type regardless of collection site was analyzed by principal component analysis of Bray-Curtis dissimilarity matrixes. P-values from PERMANOVA analysis are as follows (A) p-value = 0.005, (B) p-value = 0.001, (C) p-value = 0.001, (D) p-value = 0.001, (E) p-value = 0.001.
(EPS)

S8 Fig The percent abundance of the most abundant genera is plotted by container type (coconut and plastic) and collection site (Site 1, Site 2, and Site 3) and separated out by individual larvae. (EPS)

S9 Fig The log2fold change of bacteria genera with significant differences between Plastic and Coconut is plotted. Pairwise differential analysis was performed between larvae from Site 1–3 after receiving bacteria from either a coconut- or plastic-derived microbiome. Negative value indicates a higher abundance in the Plastic group compared to the Coconut group and a positive value indicates a higher abundance in the Coconut group compared to the Plastic group.
(TIF)

S10 Fig Bacterial abundance in L2 larvae collected from larval flasks inoculated with decreasing quantities of glycerol stock (bacteria source). Using the gnotobiotic system, larvae were reared with undiluted, a 1:10 or 1:100 dilution of the glycerol stock (bamboo shoot June 2022). On day two, larvae were collected and surface sterilized before manual homogenization. CFU/ml was determined by counting CFU on LB agar plates. Graph shows the log_10_ transformed CFU/ml for 3 pools of 3 individual larvae from each larval flask. Bar represents the mean. Bacterial abundance was compared by one-way ANOVA (F = 23.41, p-value = 0.0015) with Tukey’s multiple comparison tests (undiluted vs. 1:10: p-value = 0.0416; undiluted vs. 1:100: p-value = 0.0012; 1:10 vs. 1:100: p-value = 0.0258).
(TIF)

S11 Fig Abundance of bacterial taxa in larvae exposed to plastic or bamboo-derived microbiomes. The percent abundance of the 20 most abundant genera is plotted by container type (bamboo and plastic collected August 2021) and separated out by individual larvae.
(EPS)

S12 Fig Quantification of bacteria in additional preserved microbiome sources. 16S rRNA gene amplification by qPCR was done to determine the copy number in the preserved glycerol stock from a wagon, a bamboo shoot and the diluted bamboo shoot. An E. coli standard curve was used to calculate the copy number for the unknown samples. Graph represents the mean Ct for two experiments converted to Log_10_ 16S gene copy number per µl. Statistical significance was determined by one-way ANOVA (Bamboo vs. Wagon: p-value = 0.015; Wagon vs. Dil. Bamboo: p-value = 0.1823; Bamboo vs. Dil. Bamboo: p-value = 0.0524).
(TIF)

S13 Fig Community structure of all larvae and water samples compared to their respective negative controls. Beta diversity metrics were analyzed by sample type **(A)** larvae and (B) water against their negative controls by principal component analysis of Bray-Curtis dissimilarity matrixes at the OTU level. P-values from PERMANOVA analysis are as follows (**A**) p-value = 0.001, (**B**) p-value = 0.063.
(EPS)

S1 Table Bloodmeal titers, associated ZIKV isolate, and mosquito line organized by figure. (XLSX)

S2 Table Mock breeding site locations. Generalized location and type of environment for each collection site where containers were placed to gather rainwater.
(XLSX)

S3 Table Statistical analysis output. (XLSX)

S4 Table Bacterial abundance in larval flasks over time. (XLSX)

S5 Table Raw data. (XLSX)
