# Supplementary figures and images for: Reduced microbe abundance in an urban larval development container increases Aedes aegypti susceptibility to Zika virus
Source: PLoS Pathog. 2025 May 19;21(5):e1013154. doi: 10.1371/journal.ppat.1013154 (PMC12121923; doi:10.1371/journal.ppat.1013154)

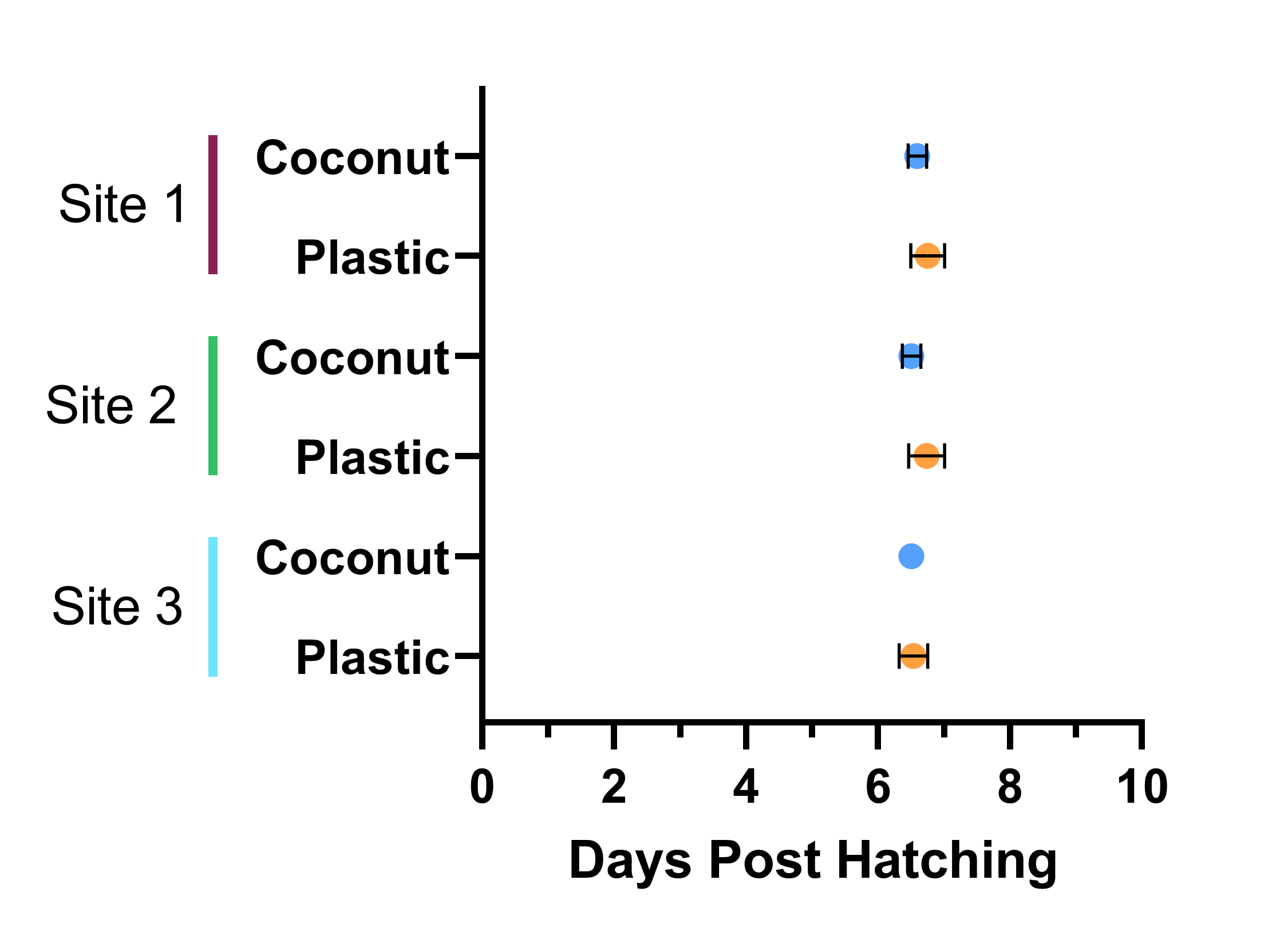

Supplement: S2 Fig — Variation in pupation rate are shown for Ae. aegypti reared in the presence of bacteria from a container-associated preserved glycerol stock in a gnotobiotic system. Graph represents the average day on which 50% of the larvae had pupated (PD50) for each site and container type determined using a simple logistic regression for each flask. Pupation rate was determined by daily pupae counts in three or five flasks (in one experiment a flask was lost for Site 1 Plastic reducing the number of flasks to two) of gnotobiotic larvae in two independent experiments. Flasks contained an average of 159 larvae. Axenic larvae included in each independent experiment failed to pupate. Statistical significance of the PD50 was assessed by one-way ANOVA (p-value = 0.9108) with Tukey’s multiple comparison tests (p-values can be found in S2 Table). Error bars represent SEM. (TIF) [file ppat.1013154.s002.tif]

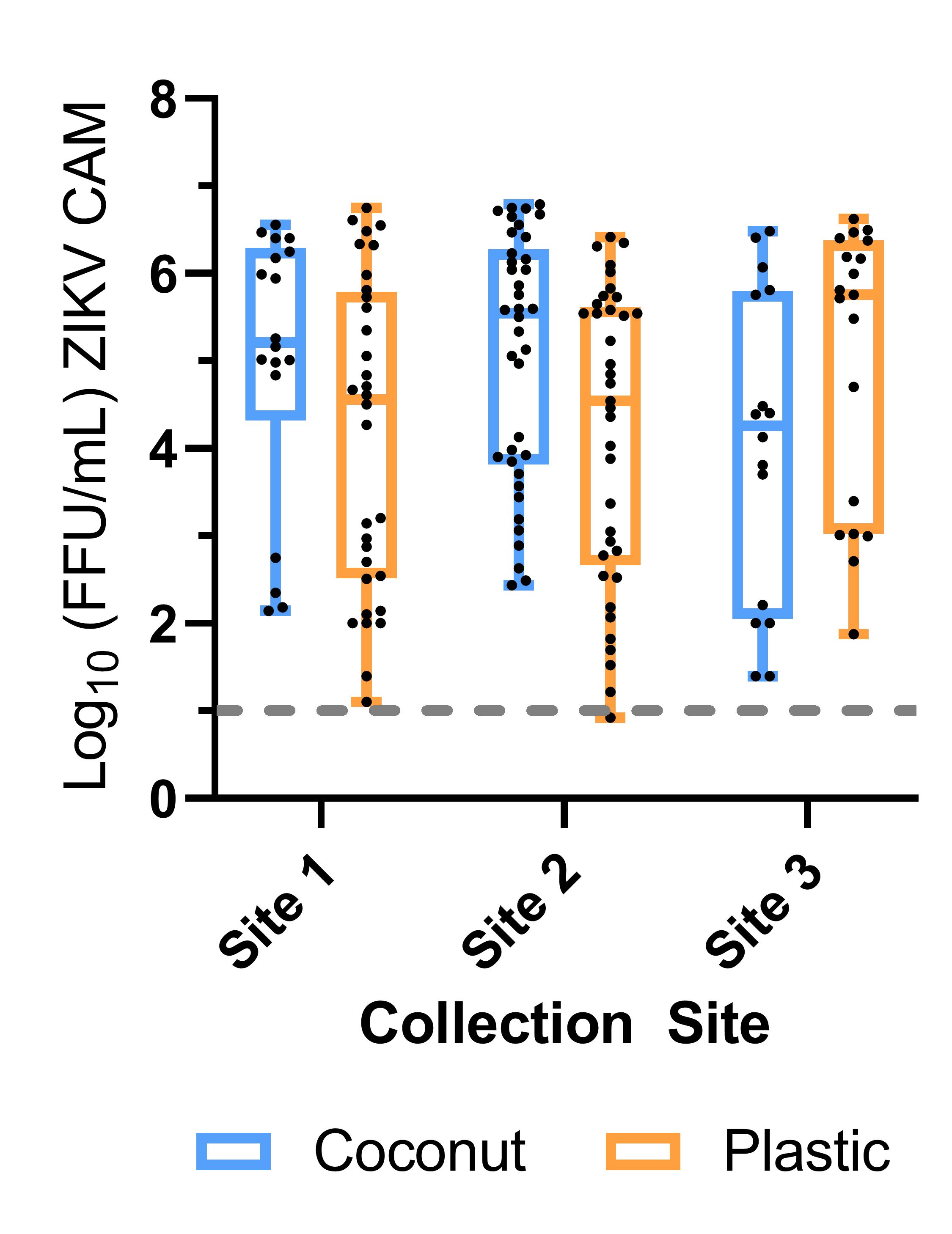

Supplement: S3 Fig — Larval development in container-derived microbiomes does not alter disseminated viral titer in a site or container specific way. Boxplot showing the dissemination titers of infectious ZIKV particles expressed as the Log10-transformed number of focus-forming units (FFU) per ml detected in the Ae. aegypti head fourteen days post-infectious blood meal (Fig 3, Dose III). Each point represents an individual and the mean is represented by a horizontal line. The error bars represent the min and max. Data represent two independent experiments expect for Site 1 coconut which has one. Data were analyzed by Two-way ANOVA as a function of container type, collection site, and their interaction (container type: p-value = 0.2503, collection site: p-value = 0.8849, container type x collection site: p-value = 0.0160). The number of positive individual mosquitoes represented is Site 1 Coconut: 18; Site 1 Plastic: 32; Site 2 Coconut: 38; Site 2 Plastic: 37; Site 3 Coconut: 16; Site 3 Plastic: 19. (TIF) [file ppat.1013154.s003.tif]

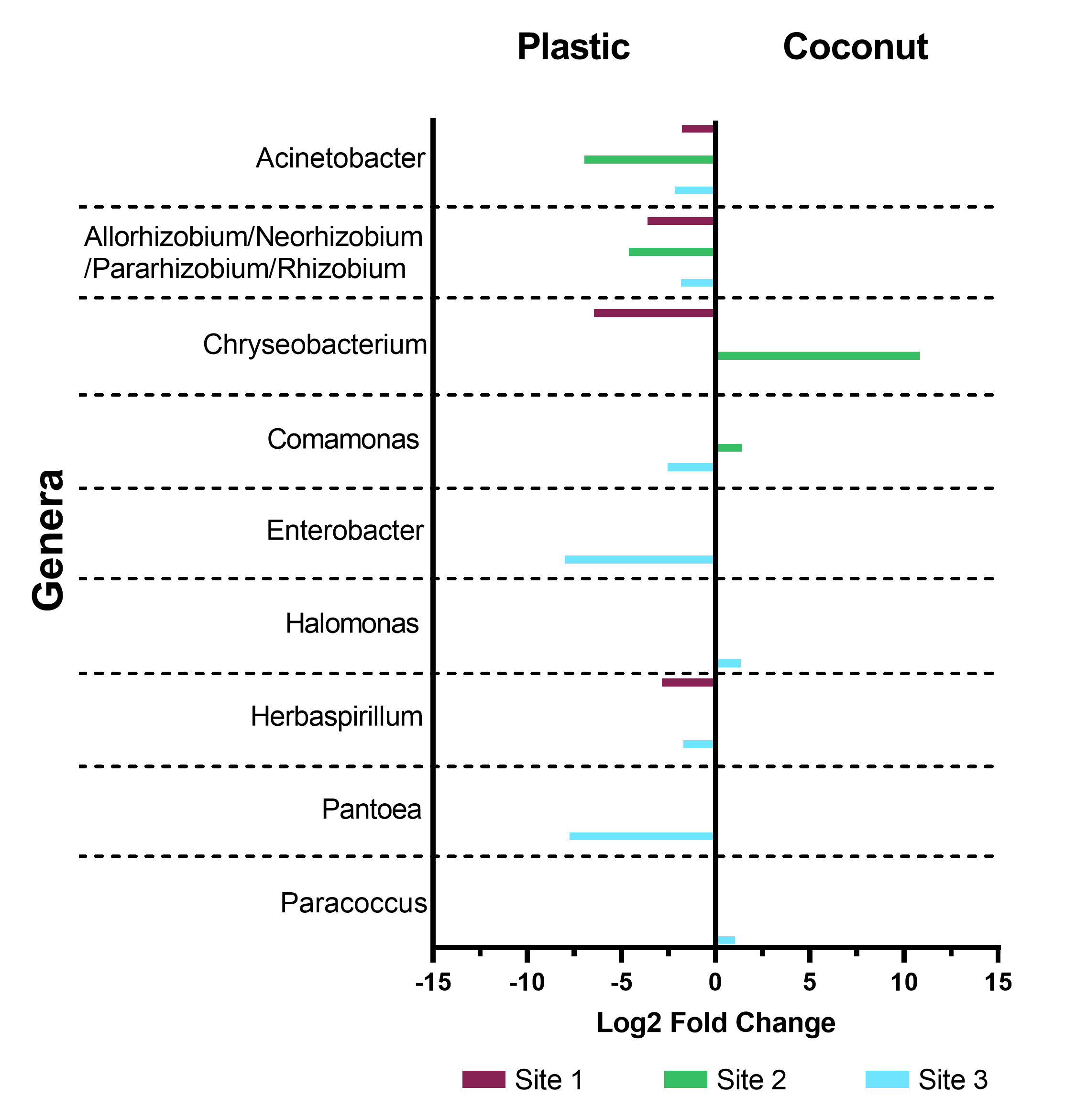

Supplement: S9 Fig — Pairwise differential analysis was performed between larvae from Site 1–3 after receiving bacteria from either a coconut- or plastic-derived microbiome. Negative value indicates a higher abundance in the Plastic group compared to the Coconut group and a positive value indicates a higher abundance in the Coconut group compared to the Plastic group. (TIF) [file ppat.1013154.s009.tif]

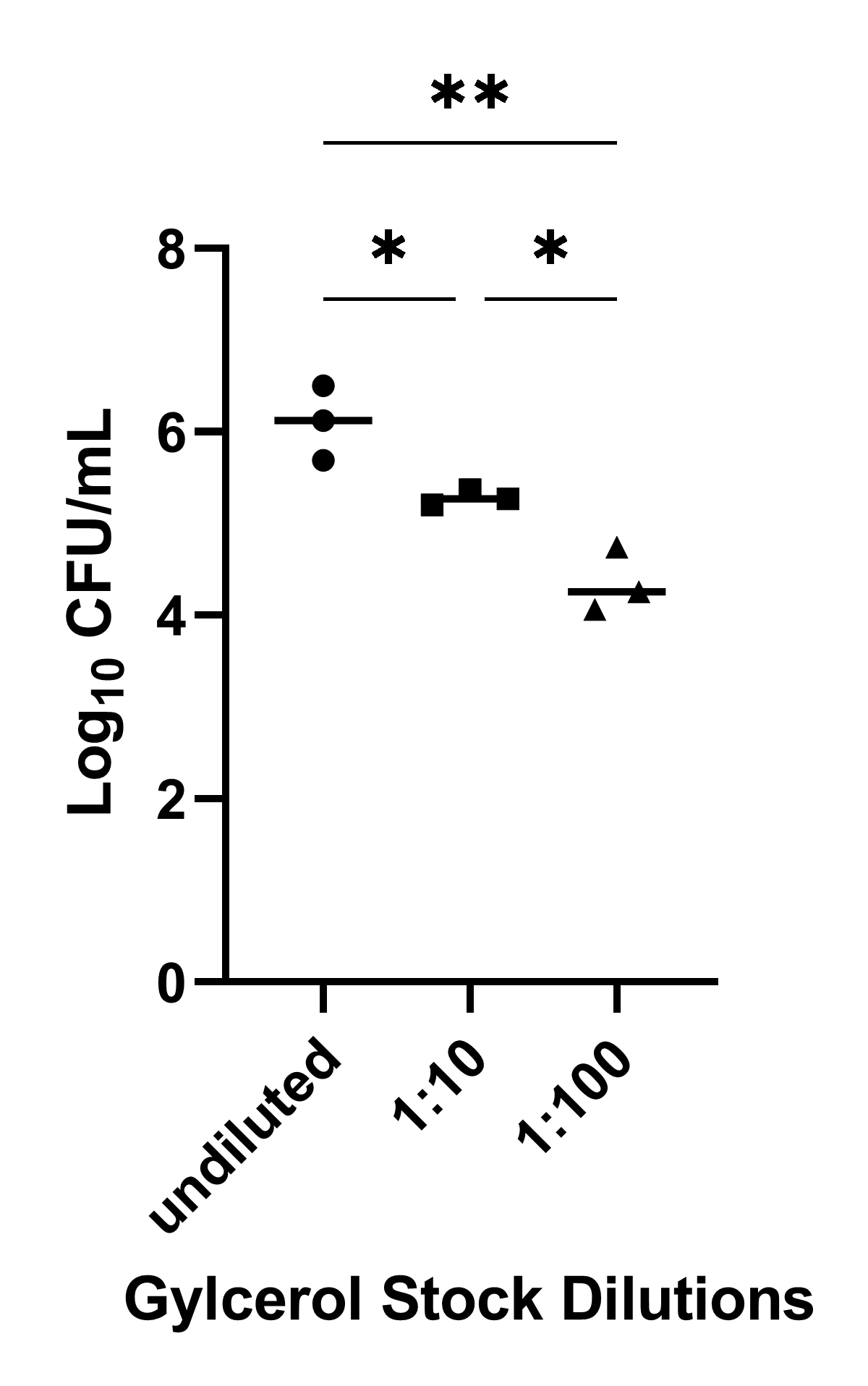

Supplement: S10 Fig — Using the gnotobiotic system, larvae were reared with undiluted, a 1:10 or 1:100 dilution of the glycerol stock (bamboo shoot June 2022). On day two, larvae were collected and surface sterilized before manual homogenization. CFU/ml was determined by counting CFU on LB agar plates. Graph shows the log10 transformed CFU/ml for 3 pools of 3 individual larvae from each larval flask. Bar represents the mean. Bacterial abundance was compared by one-way ANOVA (F = 23.41, p-value = 0.0015) with Tukey’s multiple comparison tests (undiluted vs. 1:10: p-value = 0.0416; undiluted vs. 1:100: p-value = 0.0012; 1:10 vs. 1:100: p-value = 0.0258). (TIF) [file ppat.1013154.s010.tif]

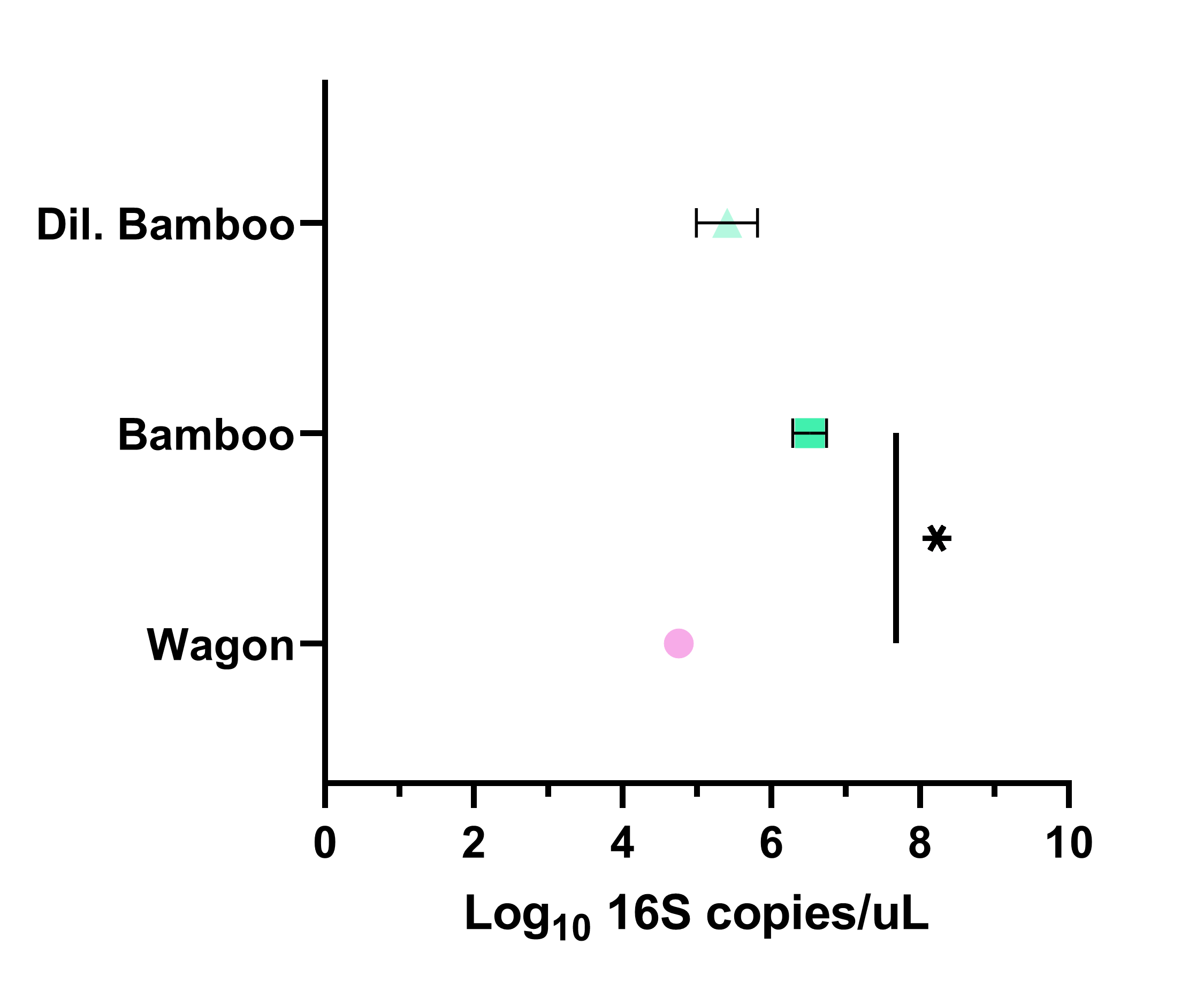

Supplement: S12 Fig — 16S rRNA gene amplification by qPCR was done to determine the copy number in the preserved glycerol stock from a wagon, a bamboo shoot and the diluted bamboo shoot. An E. coli standard curve was used to calculate the copy number for the unknown samples. Graph represents the mean Ct for two experiments converted to Log10 16S gene copy number per µl. Statistical significance was determined by one-way ANOVA (Bamboo vs. Wagon: p-value = 0.015; Wagon vs. Dil. Bamboo: p-value = 0.1823; Bamboo vs. Dil. Bamboo: p-value = 0.0524). (TIF) [file ppat.1013154.s012.tif]
